# Corneal Endothelial Cell Density and Morphology in Healthy Turkish Eyes

**DOI:** 10.1155/2014/852624

**Published:** 2014-02-10

**Authors:** Ceyhun Arıcı, Osman Sevki Arslan, Funda Dikkaya

**Affiliations:** Department of Ophthalmology, Cerrahpasa School of Medicine, Istanbul University, Olgunlar Sokak No. 6/10, Bakirkoy, 34144 Istanbul, Turkey

## Abstract

*Purpose*. To describe the normative values of corneal endothelial cell density, morphology, and central corneal thickness in healthy Turkish eyes. *Methods*. Specular microscopy was performed in 252 eyes of 126 healthy volunteers (M : F, 42 : 84). Parameters studied included mean endothelial cell density (MCD), mean cell area (MCA), coefficient of variation (CV) in cell size, percentage of hexagonal cells, and central corneal thickness (CCT). *Results*. The mean age of volunteers was 44.3 ± 13.5 (range, 20 to 70) years. There was a statistically significant decrease in MCD (*P* < 0.001; correlation, −0.388) and percentage of hexagonal cells, (*P* < 0.001; correlation, −0.199) with age. There was also a statistically significant increase in MCA (*P* < 0.001; correlation, 0.363) with increasing age. There was no statistically significant difference in MCD, MCA, CV in cell size, percentage of hexagonal cells, and CCT between genders and there was also no significant difference in these parameters between fellow eyes of subjects. *Conclusions*. Normotive data for the endothelium in the Turkish population are reported. Endothelial cell density in the Turkish eyes is less than that described in the Japanese, American, Chinese, and Filipino eyes and higher than that described in Indian, Thai, and Iranian eyes.

## 1. Introduction

Corneal endothelium is essential for the maintenance of the optical transparency of the cornea. Extrinsic factors, such as genetics, race, and age, [1–3] or intrinsic factors, such as trauma, intraocular surgery, ultraviolet radiation, and infection [4–6] are responsible for maintaining the structural and functional integrity of the corneal endothelium.

Clinical observations indicate that an endothelial cell density of 400 to 600 cells/mm^2^ is a crucial point at which endothelial decompensation develops [7]. Therefore, ECD is clinically a very important parameter. The metabolic function of these cells is also important because a reduced number of healthy endothelial cells may maintain corneal deturgescence better than a similar number of poorly functioning cells. Because proliferation of human corneal endothelial cells does not continue throughout a person's lifetime, wound healing in human corneal endothelium is mainly accomplished by cell spreading, resulting in increased cellular pleomorphism, and a decrease in the percentage of hexagonal cells with age [3, 7]. These parameters, therefore, provide an index of the functional capacity of the endothelium. Normative data regarding endothelial cell density and morphology are thus important because they facilitate assessment of the functional reserve of the endothelium in individual patients.

Due to the existence of variations in endothelial parameters in Indian, Filipino, Iranian, Thai, Chinese, Japanese, and American populations [2, 8–12], knowledge of normative data on the corneal endothelium is important. Data gathered may help in the assessment of the functional endothelial reserve of individuals and may even aid in understanding corneal disease in people of different genders, ages, and ethnic groups.

To the best of our knowledge, such data are not available in the Turkish population, which may differ from those in other races. This prospective study aimed to describe the endothelial cell characteristics and central corneal thickness in healthy Turkish eyes in regard to age and gender and reports the rate of endothelial cell loss with increasing age.

## 2. Patients and Methods

The study population comprised 126 healthy volunteers randomly selected from the visitors, outpatients, and staff of Istanbul University, Cerrahpasa Medical Faculty, Department of Ophthalmology. Subjects enrolled in the study signed an informed consent form, and the study was in accordance with the tenets of the Helsinki Declaration.

### 2.1. Selection Criteria

Only healthy volunteers with ages between 20 and 70 years old and with best-corrected visual acuity at least 1,0 (on the Snellen scale) for both eyes, refractive error (in spherical equivalent) within ±2.00 diopters, were enrolled in the study. Exclusion criteria included history of intraocular surgery or ocular trauma, corneal opacity, glaucoma, uveitis, evidence of endothelial dystrophy on slit-lamp biomicroscopy, family history of corneal decompensation, use of contact lens, and diabetes mellitus.

### 2.2. Examinations

Routine ocular examination was performed, and if the participant was found to be suitable for the study, corneal endothelial cell density, morphology and central corneal thickness were examined with noncontact specular microscopy (SP-3000P: Topcon corporation, Tokyo, Japan). A single examiner performed all measurements between 10:00 and 11:30 am. The procedure for specular microscopy was as follows: three images from central cornea were taken and at least 100 contiguous cells and were manually marked by the examiner for analysis by a built-in software program.

Parameters recorded from the system included mean endothelial cell density (MCD) (cell/mm^2^), mean cell area (MCA) (*μ*m^2^), coefficient of variation (CV) in cell size, percentage of hexagonal cells, and central corneal thickness (CCT). The CV in cell size (standard deviation divided by the mean cell area) was used as an index of the extent of variation in the cell area (polymegathism). The percentage of hexagonal cells in the analyzed area was used as an index of variation in cell shape (polymorphism).

### 2.3. Statistical Analysis

Data analysis was performed using SPSS software (version 10.0, SPSS, Inc.). The paired and unpaired *t*-test and Pearson correlation analysis were used. Analysis of variance (ANOVA) and linear regression analysis were used to examine the change in endothelial cell characteristics with age. Data are shown as mean ± standard deviation. *P* values less than 0.05 were considered significant.

## 3. Results

The endothelial cell characteristics of 252 eyes of 126 healthy Turkish volunteers were studied. The mean age of the study population was 44.3 ± 13.5 years, the range being 20 to 70 years old. There were 42 males and 84 females. The MCD of the population was 2671 ± 356 cell/mm^2^ (range, 1834 to 3652 cell/mm^2^). The MCA was 381.2 ± 51.9 *μ*m^2^ (range, 274–545 *μ*m^2^). The mean CV in cell size was 34.3 ± 5.3 (range, 22 to 49), the mean percentage of hexagonal cells was 54.9 ± 10.0% (range, 16 to 80%), and CCT was 521 ± 33 *μ*m (range, 439 to 621 *μ*m). For the purpose of comparison, subjects were divided by decade of age, and this resulted in 5 subgroups, starting from the third decade (21–30 years; Table 1).

The endothelial cell characteristics did not change significantly between males and females in different age decades (*P* > 0.05) (Table 2). There were also no statistically significant differences in the endothelial cell characteristics between fellow eyes (*P* > 0.05) (Table 3).

The rate of cell loss in each decade of life was studied (Table 4), and, in general, a gradual decrease in the rate of such parameter was noted with advancing age. The highest rate of loss was noted in the 3rd and 5th decade of life in this study population (5.9% and 5.1%, resp.).

MCD (*P* < 0.001; correlation, −0.388) and percentage of hexagonal cells (*P* < 0.001; correlation, −0.199) and CCT (*P* < 0.001; correlation, −0.241) decreased significantly with age. In addition, MCA increased significantly (*P* < 0.001; correlation, 0.363) with increasing age.

Endothelial cell counts in the study population were compared with previously described values for the Japanese, American, Chinese, Filipino, Indian, Thai, and Iranian populations (Table 5).

## 4. Discussion

Several studies have reported the relationship of endothelial cell density and morphology with age, gender, and ethnicity. It is clear that significant differences in corneal endothelial properties do exist among races and ethnic groups [2, 8–12]. Therefore, it is important for populations of different racial and ethnic backgrounds to establish normative data on which decisions regarding endothelial function can be based. We have developed the first study reporting the corneal endothelial cell characteristics and CCT in Turkish population. Our study showed that in the Turkish population with increasing age there is a general trend toward decreased MCD, increased MCA, increased CV in cell size, and a decreased percentage of hexagonal cells. Although direct comparisons of our results with the results reported by others are limited by the variations in the method used to study the endothelial characteristics, a trend toward lesser cell counts with advancing age is common to these reports after the third decade of life [2, 8, 10, 11, 13]. Our data revealed lesser cell counts compared with the values reported in Japanese, American, Chinese, and Filipino eyes; they were higher than those in Indian, Thai, and Iranian eyes [2, 8–12].

We, however, noted a variation in the cell loss rate in different age groups with a higher loss of cells at the third and fifth decades. The reason for this increased rate of cell loss at these decades is not clear, and this may be related to an increased metabolic destruction of the endothelial cells with advancing age.

Matsuda et al. [12] found that the average corneal diameter in the American eyes was larger than that in the Japanese eyes. Regarding the differences seen in their data, Matsuda et al. [12] speculated that the larger corneal surface area in American eyes could be responsible for the lesser endothelial cell density when compared with Japanese eyes. Rao et al. [8] found that the average corneal diameter in their study in healthy Indian eyes was larger than that in the normal American and Japanese eyes and MCD was lower than that in the American and Japanese eyes. Once we did not measure the corneal diameter, we are unable to perform a similar analysis.

It has been reported that positive correlation between CCT and MCD is controversial [8, 14]. Our results showed that the CCT tends to be thinner with age.

In conclusion, this study reports normative endothelial characteristics in a Turkish population that may serve as a useful baseline for future studies. This study has reported a consistent decrease in MCD and percentage of hexagonal cells, increase in MCA and CV in cell size with age. There was no statistically significant difference in endothelial cell charecteristics and CCT between genders. There was also no significant difference in these parameters between fellow eyes of subjects. Comparison with previous studies indicates that endothelial cell density in the Turkish eyes is less than that reported in the Japanese, American, Chinese, and Filipino eyes, However, the value for Turkish is higher than that reported in Indian, Thai, and Iranian eyes.

## Figures and Tables

**Table 1:** Endothelial cell characteristics of the study population in different age groups.

Age group (yr)	Age (yr) (mean ± SD)	Number of eyes	Cell density (cell/mm²) (mean ± SD)	Cell area (*μ*m²) (mean ± SD)	CV in cell size (%) (mean ± SD)	Hexagonality (%) (mean ± SD)	CCT (*μ*m) (mean ± SD)
20–30	23.3 ± 3.1	42	2910.2 ± 365.9	349.3 ± 46.5	30.5 ± 4.0	60.2 ± 9.4	534.5 ± 32.6
31–40	35.4 ± 3.2	54	2738.3 ± 389.4	373.0 ± 56.7	34.9 ± 5.4	55.9 ± 9.7	520.1 ± 31.4
41–50	45.2 ± 2.6	58	2682.0 ± 286.7	377.3 ± 42.2	36.0 ± 4.9	52.8 ± 11.2	530.4 ± 30.9
51–60	52.8 ± 2.7	56	2546.0 ± 276.4	397.4 ± 44.1	35.2 ± 6.0	52.6 ± 8.9	509.9 ± 32.6
61–70	64.1 ± 3.6	42	2497.6 ± 331.7	407.2 ± 53.3	33.6 ± 3.8	54.4 ± 8.7	513.1 ± 30.8

**Table 2:** Endothelial cell characteristics in female and male.

Age group (yr)	Female (mean ± SD)	Male (mean ± SD)	*P* value
20–30			
Cell density (cell/mm^2^)	2981.9 ± 396.9	2858.2 ± 372.8	0.487
Cell area (*μ*m^2^)	341.3 ± 49.1	355.0 ± 45.1	0.531
CV in cell size (%)	30.8 ± 4.1	29.5 ± 3.3	0.461
Hexagonality (%)	59.4 ± 11.2	59.3 ± 11.1	0.979
CCT (*μ*m)	540.0 ± 37.5	521.3 ± 25.1	0.228
31–40			
Cell density (cell/mm^2^)	2663.1 ± 329.9	2852.4 ± 450.4	0.233
Cell area (*μ*m^2^)	381.5 ± 51.1	359.5 ± 64.9	0.355
CV in cell size (%)	35.1 ± 5.7	35.7 ± 5.3	0.784
Hexagonality (%)	55.2 ± 9.5	59.1 ± 10.5	0.353
CCT (*μ*m)	522.4 ± 31.7	514.6 ± 29.8	0.558
41–50			
Cell density (cell/mm^2^)	2981.9 ± 396.9	2858.2 ± 372.8	0.487
Cell area (*μ*m^2^)	383.6 ± 35.2	372.8 ± 47.6	0.488
CV in cell size (%)	34.7 ± 4.5	35.8 ± 4.7	0.522
Hexagonality (%)	52.3 ± 12.2	53.3 ± 12.8	0.840
CCT (*μ*m)	536.6 ± 27.8	523.9 ± 35.5	0.290
51–60			
Cell density (cell/mm^2^)	2525.4 ± 279.8	2613.0 ± 251.9	0.495
Cell area (*μ*m^2^)	401.0 ± 47.8	385.6 ± 36.5	0.474
CV in cell size (%)	35.1 ± 6.5	36.4 ± 4.5	0.646
Hexagonality (%)	54.7 ± 9.0	46.3 ± 8.5	0.051
CCT (*μ*m)	511.4 ± 34.3	511.8 ± 27.8	0.978
61–70			
Cell density (cell/mm^2^)	2409.4 ± 271.4	2592.1 ± 360.5	0.201
Cell area (*μ*m^2^)	420.0 ± 47.8	392.7 ± 57.5	0.254
CV in cell size (%)	32.4 ± 4.0	32.8 ± 5.5	0.831
Hexagonality (%)	53.8 ± 9.2	55.8 ± 5.9	0.595
CCT (*μ*m)	519.5 ± 32.0	507.4 ± 33.4	0.415

**Table 3:** Corneal endothelial characteristics in right and left eyes of subjects.

Age group (yr)	Right eye (mean ± SD)	Left eye (mean ± SD)	*P* value
20–30			
Cell density (cell/mm^2^)	2934.8 ± 383.3	2885.6 ± 355.4	0.755
Cell area (*μ*m^2^)	346.5 ± 47.0	352.0 ± 47.1	0.708
CV in cell size (%)	30.3 ± 3.8	30.8 ± 4.2	0.684
Hexagonality (%)	59.3 ± 10.9	61.0 ± 7.9	0.669
CCT (*μ*m)	532.9 ± 33.9	536.1 ± 31.9	0.572
31–40			
Cell density (cell/mm^2^)	2719.2 ± 371.1	2757.4 ± 413.0	0.997
Cell area (*μ*m^2^)	375.0 ± 55.2	371.1 ± 59.1	0.804
CV in cell size (%)	35.3 ± 5.5	34.5 ± 5.5	0.580
Hexagonality (%)	56.4 ± 9.8	55.3 ± 9.7	0.722
CCT (*μ*m)	520.1 ± 30.8	520.1 ± 32.6	0.698
41–50			
Cell density (cell/mm^2^)	2665.7 ± 274.1	2698.4 ± 302.7	0.821
Cell area (*μ*m^2^)	379.1 ± 40.3	375.4 ± 44.7	0.741
CV in cell size (%)	35.1 ± 4.6	36.9 ± 5.1	0.172
Hexagonality (%)	52.7 ± 12.2	52.9 ± 10.3	0.669
CCT (*μ*m)	531.3 ± 31.2	529.5 ± 31.1	0.954
51–60			
Cell density (cell/mm^2^)	2544.2 ± 272.0	2547.8 ± 285.7	0.716
Cell area (*μ*m^2^)	397.7 ± 45.4	397.1 ± 43.5	0.964
CV in cell size (%)	35.4 ± 6.0	35.0 ± 6.0	0.814
Hexagonality (%)	52.9 ± 9.4	52.4 ± 8.6	0.961
CCT (*μ*m)	511.5 ± 32.5	508.3 ± 32.5	0.836
61–70			
Cell density (cell/mm^2^)	2479.0 ± 313.0	2516.3 ± 356.3	0.708
Cell area (*μ*m^2^)	409.6 ± 52.1	404.8 ± 55.8	0.775
CV in cell size (%)	32.6 ± 4.5	34.6 ± 2.6	0.074
Hexagonality (%)	54.5 ± 8.0	54.2 ± 9.5	0.720
CCT (*μ*m)	514.9 ± 32.3	511.3 ± 29.9	0.917

**Table 4:** Endothelial cell loss by decade of age.

Age group (yr)	Cell density (cell/mm²) (mean ± SD)	Cell loss rate (%)
21–30	2910.2 ± 365.9	
31–40	2738.3 ± 389.4	5.9
41–50	2682.0 ± 286.7	2.1
51–60	2546.0 ± 276.4	5.1
61–70	2497.6 ± 331.7	1.9

**Table 5:** Comparison of endothelial cell density in Turkish, American, Japanese, Indian, Chinese, and Iranian eyes.

Age group (yr)	Turkish		American [12]		Japanese [12]		Indian [8]		Chinese [11]		Iranian [10]	
	Number of eyes	Cell density (cell/mm^2^) (mean ± SD)	Number of eyes	Cell density (cell/mm^2^) (mean ± SD)	Number of eyes	Cell density (cell/mm^2^) (mean ± SD)	Number of eyes	Cell density (cell/mm^2^) (mean ± SD)	Number of eyes	Cell density (cell/mm^2^) (mean ± SD)	Number of eyes	Cell density (cell/mm^2^) (mean ± SD)
20–30	42	2910 ± 366	11	2977 ± 324	18	3893 ± 259	104	2782 ± 250	100	2988 ± 243	102	2407 ± 399
31–40	54	2738 ± 389	6	2739 ± 208	10	3688 ± 245	96	2634 ± 288	100	2920 ± 325	45	2245 ± 349
41–50	58	2682 ± 287	11	2619 ± 321	10	3749 ± 407	97	2408 ± 274	97	2935 ± 285	66	2071 ± 340
51–60	56	2546 ± 276	13	2625 ± 172	10	3386 ± 455	98	2438 ± 309	97	2810 ± 321	87	1939 ± 344
61–70	42	2498 ± 332	8	2684 ± 384	6	3307 ± 330	88	2431 ± 357	90	2739 ± 316	122	1775 ± 348
